# Detection of Fungus Infection on Petals of Rapeseed (*Brassica napus* L.) Using NIR Hyperspectral Imaging

**DOI:** 10.1038/srep38878

**Published:** 2016-12-13

**Authors:** Yan-Ru Zhao, Ke-Qiang Yu, Xiaoli Li, Yong He

**Affiliations:** 1 College of Biosystems Engineering and Food Science, Zhejiang University, 866 Yuhangtang Road, Hangzhou 310058, China; 2 College of Mechanical and Electronic Engineering, Northwest A&F University, Yangling, 712100, China

## Abstract

Infected petals are often regarded as the source for the spread of fungi *Sclerotinia sclerotiorum* in all growing process of rapeseed (*Brassica napus* L.) plants. This research aimed to detect fungal infection of rapeseed petals by applying hyperspectral imaging in the spectral region of 874–1734 nm coupled with chemometrics. Reflectance was extracted from regions of interest (ROIs) in the hyperspectral image of each sample. Firstly, principal component analysis (PCA) was applied to conduct a cluster analysis with the first several principal components (PCs). Then, two methods including X-loadings of PCA and random frog (RF) algorithm were used and compared for optimizing wavebands selection. Least squares-support vector machine (LS-SVM) methodology was employed to establish discriminative models based on the optimal and full wavebands. Finally, area under the receiver operating characteristics curve (AUC) was utilized to evaluate classification performance of these LS-SVM models. It was found that LS-SVM based on the combination of all optimal wavebands had the best performance with AUC of 0.929. These results were promising and demonstrated the potential of applying hyperspectral imaging in fungus infection detection on rapeseed petals.

Rapeseed (*Brassica napus* L.) is an important oil crop which is widely cultivated all around the world. High energy and protein livestock feed are mainly made from its seeds. It is also partly used as potential raw material in synthesizing biodiesel^1^. *Sclerotinia sclerotiorum (S. sclerotiorum*) is a serious and ubiquitous worldwide fungal pathogen of rapeseed plants^2^. The fungus infects leaves, stems, and pods, resulting in a tremendous loss in seed yield^3^. For example, sclerotinia stem rot of rapeseed plants in China leads to oilseed yield loss ranging from 10 to 80%, and oil quality declines as well^4^. As an important pathogen of rapeseed plants, *S. sclerotiorum* can enter leaves or stems via petals. The infection of rapeseed petals is the first step for the development of *S. sclerotiorum* on rapeseed plants because mycelium on the infected petals can penetrate into tissues of other organs^5^. A report showed that the numbers of petals and stamens sticking to rapeseed leaves are a guide to estimate the risk of leaves or stem rot status^6^. To avoid routine spraying, early diagnosis of the prevalence and severity of disease is required so that treatment can be rationalized^7^. Previously, various molecular techniques including enzyme-linked immunosorbent assay (ELISA) and polymerase chain reaction (PCR) are developed to assay the risk of fungal infection^8^,^9^,^10^. However, the disadvantages of these methods include expensive, labor-intensive, time-consuming, and tedious extraction procedures, what’s more, these detection methods mostly pointed at an individual plant, and are limited for the large-scale agricultural production^11^. Therefore, it would be beneficial if a rapid, reliable and nondestructive technique is implemented in detecting the infected petals, so as to predict the risk of disease and provide a guideline for spraying.

Spectroscopic techniques as potential method have been proved successful in detecting plants diseases^8^. Near infrared spectroscopy^12^,^13^, thermal infrared spectroscopy^14^ and Raman spectral techniques^15^,^16^,^17^ have been intensively applied to detect various diseases on plants. Unfortunately, these methods have their own limitations: spectroscopic technique is often used to measure the averaged spectrum of the sample, but is fails to provide the spectral details at each pixel on images of the targets; thermal infrared measurement often affected by the temperature of the target’s surrounding and Raman information often affected by fluorescence signals which generated in the biological tissue. Thus, hyperspectral imaging is considered as the most promising method in detecting fungal infection of plants.

Hyperspectral imaging is an innovative technology with high potential for the non-invasive sensing of the physiological status of field crops. Hyperspectral imaging is expected to improve the accuracy of disease detection through a better examination of host-pathogen interactions by measuring the pixel-wise information of disease-specific symptoms^18^. This newly developed technology has been used in an assessment of plant nutrient^19^,^20^,^21^,^22^,^23^,^24^ and plant disease^2^,^18^,^25^,^26^,^27^,^28^,^29^. Xu *et al*.^2^ developed a new Band Math-ROC (receiver operating characteristic) curve algorithm which effectively detect early lesions on oilseed rape leaves caused by *S. sclerotiorum* and *Botrytis cinerea* in the first two days after inoculation. Zhao *et al*.^25^ employed hyperspectral imaging to determine spatial distributions of chlorophyll and carotenoid contents in cucumber leaves in response to five severities of angular leaf spot (ALS) disease. Texture features combined with spectral information has a potential in detecting early blight and late blight diseases on tomato leaves^27^. Hyperspectral and thermal imaging were used to detect biotic stresses caused by fungal species on oilseed rape leaves^28^. Rumpf *et al*.^29^ applied support vector machines (SVM) and spectral vegetation indices to realize early detection and classification of various plant diseases based on hyperspectral reflectance. Mahlein *et al*.^18^ mapped spectral reflectance enabled the detection and detailed description of diseased tissue on the leaf level based on hyperspectral imaging.

Therefore, this research aim to detect fungal infection of rapeseed petals by using hyperspectral imaging for providing a reference to develop a portable detector that is capable of early diagnosis of *S. sclerotiorum* disease on rapeseed plants. In the present study, near infrared (NIR) hyperspectral imaging was firstly employed to detect fungal infection on rapeseed petals. The specific objectives were to (1) acquiring hyperspectral images of healthy and infected petals; (2) conducting cluster statistics using principal component analysis (PCA); (3) selecting optimal wavebands based on X-loadings of the first principal components (PCs) and Random frog algorithm; (4) establishing least squares-support vector machine (LS-SVM) models by full and selected wavebands; (5) employing receiver operating characteristic (ROC) curves to evaluate the performance of these discriminative models.

## Results and Discussion

### Overview of the mean spectra of rapeseed petals

**Figure 1:**
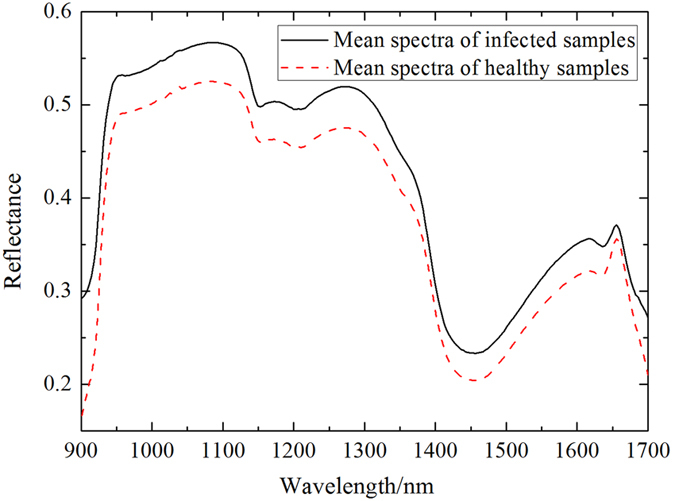
Average spectral reflectance curves of healthy and infected petals. Reflectance of healthy (n = 142) and infected (n = 105) samples were averaged and resulting two curves.

A total of 238 wavebands in the range of 900–1700 nm were selected to remove the noise at the begging and the end of wavelengths. The mean spectra of petals covering the spectral range of 900–1700 nm are presented in Fig. 1. It can be observed that there were no visibly noticeable differences between the healthy and infected petals. Some broadband peaks or valleys occurred in the NIR region in Fig. 1, it can be explained by NIR spectral region contained rich information relevant to the hydrogen containing bonds (such as O-H, C-H, and N-H)^30^. Feature valleys at 980 nm and 1450 nm (the second and first O-H stretching overtones) are connected with water. Additionally, a small valley around 1200 nm (the second C-H stretching overtone) was due to the organic matter content in samples^31^. Meanwhile, the spectral reflectance at the wavelengths of 980 nm and 1450 nm showed a sharp difference between the curves of healthy and infected petals worth nothing. What’s more, infected petals have higher reflectance than healthy samples, which might be explained by the decay phenomenon of infected petals.

Although some differences could be observed in Fig. 1, it’s impossible to estimate the infected petals directly merely based on the spectra. Thereby, chemometrics were employed to extract and concentrate the connotative information for further discriminating the healthy and infected rapeseed petals.

### Operation of PCA

**Figure 2:**
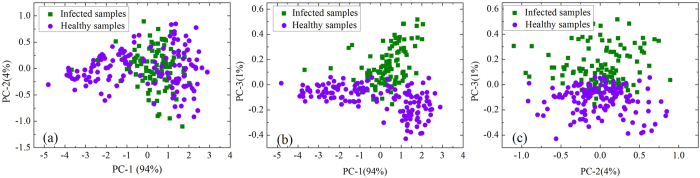
Cluster plots based on the first three PCs. (**a**) Cluster plot based on PC-1 and PC-2; (**b**) Cluster plot based on PC-1 and PC-3; (**c**) Cluster plot based on PC-2 and PC-3.

PCA was employed to transform the full wavebands (238 wavebands) into several principal components (PCs). X-loadings of first PCs were applied for qualitatively identifying the optimal wavebands that were responsible for the specific features. The first three PCs explained 99% original variations and their score plots are displayed in Fig. 2. Each point in these scatter plots represented one spectrum from one sample. Figure 2(a, b, and c) showed the clustering of two groups within the space of PC-1 and PC-2, PC-1 and PC-3, PC-2 and PC-3, respectively. In Fig. 2(a), there was a slight cross between the healthy and infected samples. In contrast, two groups of samples provided an apparent clustering in Fig. 2(b) and (c). The spectra of the infected sample were mainly scattered on the positive sides of PC-3 in Fig. 2(b) and (c), while spectra of healthy group tended to be on the negative side of PC-3 in Fig. 2(b) and (c). To sum up, the score plots distinguished the healthy and infected petals clearly.

### Selection of optimal wavebands

**Figure 3:**
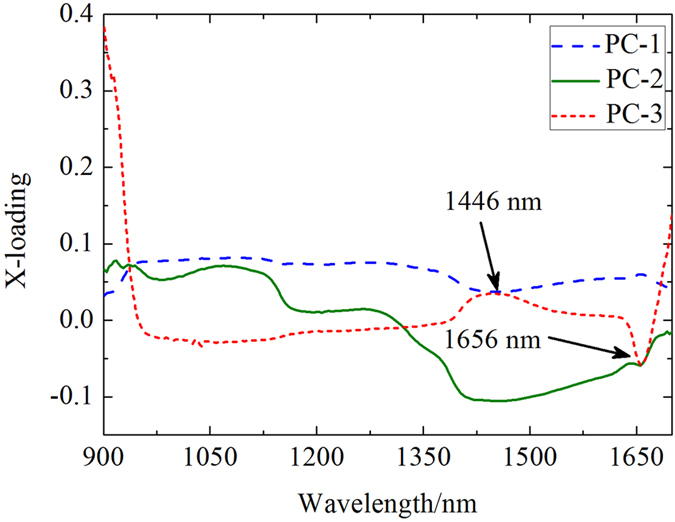
X-loadings of the PCs of the samples. 1446 and 1656 nm were selected as the optimal wavebands.

Selection of optimal wavebands is of critical significance for removing the redundant information from high-dimensional data, optimizing calibration models and producing excellent results^32^. Thus, identification of optimal wavebands carrying the most valuable and authentic information is a challenging task in the current hyperspectral data analysis. The X-loadings of PC-1 to PC-3, which revealed the importance of the analyzed variables, are shown in Fig. 3. Variable with large positive or negative loading coefficient was significant and considered as an optimal waveband. The first three loading plots of PCA indicated that the reflectance at two wavelengths of 1446 and 1656 nm with the relatively large loading coefficients had the greatest discriminatory effect on healthy and infected petals. The identified optimal waveband at 1446 nm was close to the first overtones O-H stretching near 1450 nm^31^, which implied that water content was an important factor to distinguish healthy and infected rapeseed petals.

One-way analysis of variance (ANOVA) was used to reveal the difference of the spectra between healthy and infected petals at 1446 and 1656 nm. The statistical results are shown in Table 1. The letter a, b represent the significant difference of the samples. An apparent difference between the two groups was revealed from the spectral reflectance at 1446 nm, which contributed to the different water content of healthy and infected petals. However, optimal waveband selection by PCs of X-loadings neglect the influence from categorical value of the samples.

Before RF analysis, SPXY method proposed by Galvao *et al*.^33^ was implemented to divide the spectral matrix and corresponding labeled classes into calibration sets with 165 samples (95 healthy and 70 infected samples) and prediction sets with 82 samples (47 healthy and 35 infected samples) for subsequent hyperspectral analyses.

**Figure 4:**
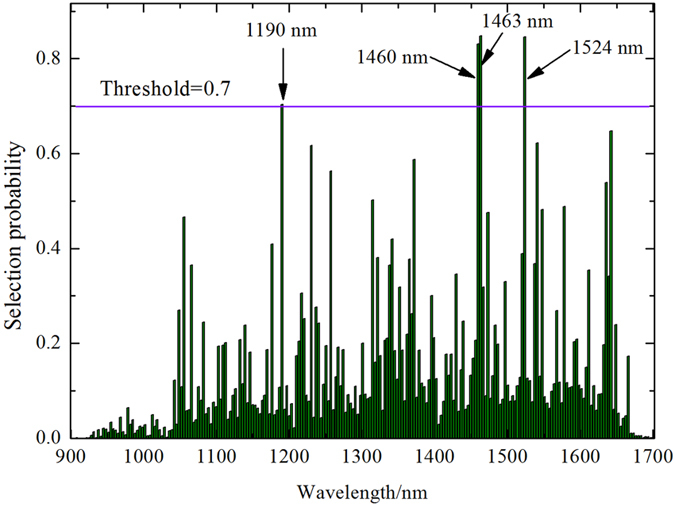
Selection probability (SP) of each wavelength averaged over 50 runs of Random frog algorithm.

RF was performed 50 times and the average value of these 50 runs was taken as the criterion for estimating the importance of each variable. The selection probability (SP) of each wavelength is shown in Fig. 4. From the SP curve, the SP of the most wavelengths offered relatively low values, whereas a small number of wavelengths exhibited extremely high SP values. Based on SP values, wavelengths with high SP (SP > 0.7) were viewed as the optimal wavelengths and labeled in the corresponding position in this study. This result indicated that the majority of wavelengths provided a weak or void relevance to the discrimination of healthy and infected rape flower samples. All the variables were ranked in descending order according to the SP value. Lastly, based on the SP curves in Fig. 4 (threshold value was set as 0.7), four wavelengths at 1190, 1460, 1463, and 1524 nm were selected as optimal wavelengths for infected petals discrimination. The selected optimal wavebands of 1460 and 1463 nm were assigned to first overtones O-H stretching around 1450 nm. The optimal waveband of 1190 nm was close to the second overtones of C-H stretching near 1210 nm^34^,^35^. The remaining selected optimal waveband were ascribed to the absorption peak of N-H group in the biological tissue^36^. Different optimal wavebands were identified by different selection methods for a different feature selection criterion of each method.

### Establishment and evaluation of the LS-SVM model

LS-SVM methodology was employed to establish models based on full wavebands (Full-LS-SVM), optimal wavebands (1190, 1460, 1463, and 1524 nm) selected by RF (RF-LS-SVM), optimal wavebands identified by X-loadings of PCs (XL-LS-SVM), a combination of optimal wavebands from RF and X-loadings (RX-LS-SVM), and an important wavelength (1446 nm-LS-SVM) in detecting the infected rapeseed petals, respectively. Recognition rate (RR) of each classification model are presented in Table 2. Results showed that the Full-LS-SVM has an accuracy of 100%, nevertheless, it is not suitable for real-time assessment due to the mass data. The XL-LS-SVM with only two optimal wavelengths (99.16% variables were removed) provided almost the same accuracy with the RF-LS-SVM based on four optimal wavelengths (98.31% variables were removed), hence, the XL-LS-SVM model had better performance than the RF-LS-SVM classification model. The RX-LS-SVM with RR of 92.68% had the best classification result than other models, meanwhile, 97.48% (6 vs. 238) variables were eliminated in the optimal wavebands selection process. Although there was significant difference in healthy and infected rapeseed petals at 1446 nm, the 1446-LS-SVM with RR of 69.51% was unsatisfactory to detect infected petals. In conclusion, the RX-LS-SVM discriminative with the combination of optimal wavelengths provided the highest RR in detecting fungus infection on rapeseed petals.

**Figure 5:**
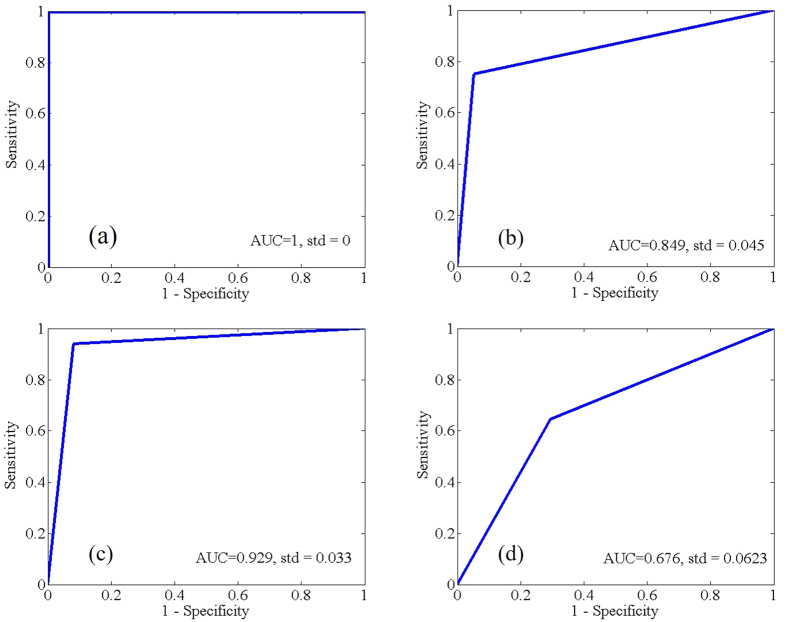
ROC curves of LS-SVM models based on (**a**) full wavelengths in the region of 900–1700 nm (238 wavebands); (**b**) optimal wavelengths at 1190, 1460, 1463, 1524 nm, and 1446, 1656 nm; (**c**) combination optimal wavebands of random frog and X-loadings (**d**) single wavelengths at 1446 nm.

After that, receiver operating characteristics (ROC) curve was employed to evaluate the performance of these LS-SVM models. ROC curves of the three the LS-SVM discriminant models were shown in Fig. 5, AUC in the Full-LS-SVM (Fig. 5a), RF-LS-SVM (Fig. 5b), XL-LS-SVM (Fig. 5b), RX-LS-SVM (Fig. 5c) and 1446-LS-SVM (Fig. 5d) models were 1, 0.849, 0.849, 0.929, and 0.676, respectively. According to the discriminate criterion of AUC^37^, the Full-LS-SVM has excellent performance in discriminating the infected petals, however, there were 238 wavelengths involved in the modelling, which would slow down the computing speed. By contrast, the RX-LS-SVM with only six optimal wavebands provided excellent classification in detecting the infected petals. Although there was only one waveband in the 1446-LS-SVM model, the AUC of 0.676 displayed in this model is sufficient to detect the infected rapeseed petals. What’s more, most of the optimal wavebands selected by RF and X-loading were related with water molecule^35^. The results showed that water content in petals is the dominant factor in discriminating the infected rapeseed petals.

Usually, infection via young and healthy living petals is clearly of primary importance in the establishment of *S. sclerotiorum* on rapeseed, therefore, detection of the infected petals would be of great value in estimating the level of disease risk and recommending of fungicide applications^7^. Infected petals often fell off from the stamen and covered on the leaves or stems of oilseed rape plants, counting the number of fallen petals on oilseed rape leaves or stems by the rapid method is important for the disease assessment. In addition, rapeseed petals are generally infected by ascospores. Scholars have already applied PCR^38^ to detect *S. sclerotiorum* on petals before their fall. Therefore seeking a fast and invasive detection method in detecting ascospores on petals is our future challenge.

## Conclusion

This study provides a theoretical basis in detecting fungal infected rapeseed petals with NIR hyperspectral imaging technique and chemometrics. PCA was employed to process cluster analysis of the healthy and infected samples based on the first three PCs. 1446 nm and 1656 nm were extracted according to the X-loadings of the PC-1, PC-2, and PC-3. Meanwhile, one-way analysis of variance (ANOVA) was used to analyze the significant difference of the reflectance at the two wavelengths. Four wavelengths at 1190, 1460, 1463, and 1524 nm were selected as optimal wavelengths to detect the infected petals. ROC showed that LS-SVM established by the six combination optimal wavebands (1190, 1460, 1463, 1524, 1446 and 1656 nm) provided the optimal results to estimate the infection petals. Applying optimal wavelengths to detect fungus infection on rapeseed petals is critical for sensor’s real-time assessment.

## Materials and Methods

### Samples

**Figure 6:**
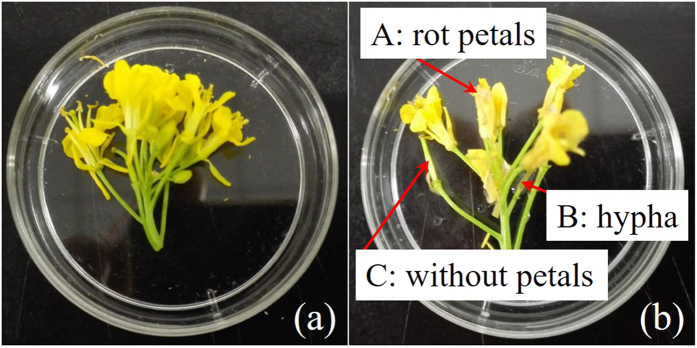
Experimental samples in petri dishes (**a**) healthy flower, petals were intact (**b**) infected flowers, A: petals were rot; B: petals fell off from stamen; C: petals were covered with some mycelium.

In this study, *Sclerotinia sclerotiorum* were cultured on PDA (Potato dextrose agar) medium, forming mycelial blocks of 5 mm in diameter^2^. Mycelial blocks were placed onto the rapeseed petals. Fresh flowers of rapeseed plants were collected from an experimental field on April 21st, 2016 at Zhejiang University (120°09′E, 30°14′N), China. All samples were placed in the same condition with constant temperature 25 °C and humidity 85%, some flowers were placed with the hypha and the rest were placed with the agar block as controlled group. Finally, 142 healthy flowers and 105 infected samples were selected from 24 h after inoculation with hypha and agar block by experienced technical personnel. The experimental samples are shown in Fig. 6, all the flowers had similar color, however, the petals of healthy flower were extended and intact, while, the infected petals decayed with water-soaked, some mycelium covered on the petals and petals fell off from the stamen.

### Hyperspectral imaging system and images acquisition

A laboratory hyperspectral imaging equipment, which covers the wavelength of 874–1734 nm with 256 wavebands, was used to capture the hyperspectral images of samples with reflectance mode. It consists of a mobile transporter operated by a stepper motor (IRCP0076, Isuzu Optics Crop, Taiwan, China), an assembled illumination with two 150-W quartz tungsten halogen lamps (Fiber-Lite DC950 Illuminator; Dolan Jenner Industries Inc, Boxborough, MA, USA), an imaging spectrograph (ImSpector N17E, Spectral Imaging Ltd., Specim, Finland) with a spectral resolution of 5.0 nm from 874–1734 nm, a CCD camera (C8484–05, Hamamatsu city, Japan) with a zoom lens (OLES23, Specim, Spectral Imaging Ltd., Oulu, Finland), and the area CCD array detector has 372 pixels × 256 bands (spatial × spectral) and operation of these equipment were controlled by a computer. The whole device (except the computer) was fixed in a dark chamber to minimize the effects of ambient light during the sample scanning.

Some parameters needed to be adjusted to acquire clear and undistorted hyperspectral images before scanning samples. Illumination unit should set an appropriate intensity and adjust a proper angel to make the light gather in a linear area of the conveyor belt just below the imaging spectrograph. The distance between samples and lens was 220 mm. All samples placed on the conveyor belt and moved at a speed of 17 mm/s to be scanned with an exposure time of 4 ms during the image acquisition.

Because the existence of dark current in CCD camera and the uneven intensity of illumination in different bands, some bands with weaker light intensity contained bigger noises^25^. Hence, the raw hyperspectral images (*I*_*0*_) required to be calibrated and the calibration process could be finished using the following Equation (1)^39^:


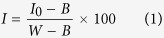


where, *I* were the calibrated hyperspectral images of the samples;

*B* were the dark reference images obtained with light source off;

*W* were the white reference images acquired from a white reference ceramic tile. Calibration is the essential for subsequent processing and analysis.

**Figure 7:**
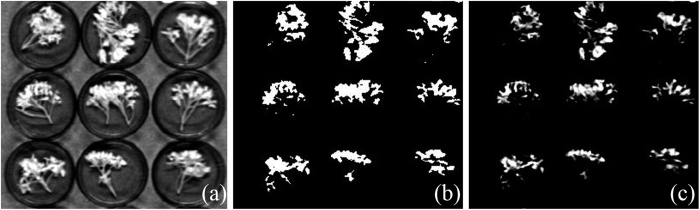
Procedure of image segmentation for subsequent extracting spectral reflectance. (**a**) a gray scale image at 1146 nm; (**b**) petal mask; (**c**) petal hyperspectral images without background.

The precondition for extracting the spectral reflectance was by identifying the regions of interest (ROIs) on petals of rapeseed. Based on the irregular and complex shape of rape flower, image segmentation was used to isolate hyperspectral images of rapeseed flowers from background and stem information, and the main process is shown in Fig. 7. There were big differences among the spectrum (peak or peak shape) of petals, stems, and backgrounds based on the pre-analysis. To isolate petals from the stem and background in hyperspectral images, the single band image at 1146 nm (Fig. 7a) with the biggest contrast was selected to process the image segmentation using a threshold (here is 0.5). The resulting segmented image in Fig. 7(b) was considered as the mask to remove the background information. By masking every hyperspectral image with the rape flower mask, the target region of petal hyperspectral images is displayed in Fig. 7(c).

The segmented region was considered as the region of interest (ROI) of the corresponding petal sample. The spectra of each pixel in ROI were averaged and this spectrum was considered as the spectral reflectance of a sample. The spectra of other samples were obtained in the same way as mentioned above. This process was finished in ENVI 4.6 software (ITT visual information solutions, Boulder, CO, USA).

### Data analysis

Principal component analysis (PCA) is an unsupervised technique (classes or composition of the samples in the data matrix is not involved) and highly popular in extracting feature and reducing the dimension of multivariate data sets. Mathematically, several principal components (PCs), which are orthonormal and can explain maximum variance, are produced in PCA transform^40^. Each PC is a linear sum of variables multiplied by corresponding weighting coefficients. Generally, most of the relevant information in initial variables (spectra) can be explained by first several PCs. Meanwhile, the score of PCs is used to reveal the features of variable distribution, and the X-loadings of PCs can exhibit the importance of different variables^41^.

Random frog (RF) methodology, a novel and efficient technique for variable selection, was carried out to identify optimal wavebands. In principle, RF borrows the framework of reversible jump Markov Chain Monte Carlo (RJMCMC) methods and employed to perform feature extraction for selecting a series of variables which describe the correlation between the predictor variables and the response variables^42^. In the interior of RF algorithm, partial least squares-linear discriminant analysis (PLS-LDA) is conducted as a modeling method. The relationship between spectral matrix (*n *× *p*) consisting of *n* samples and *p* variables and property matrix (*n *× 1) is established. RF works in an iterative manner. The algorithm of RF was fully detailed in literatures^42^,^43^. Generally, RF is implemented many times (depending on the data) to weaken the influence caused by Monte Carlo strategy embedded in RF algorithm.

Least-squares support vector machine (LS-SVM), an optimized version of the standard SVM, is a powerful methodology for pattern recognition and function estimation. It could reduce the complexity of the calculation and shorten the needed time to improve the studying ability of high-dimensional characteristic space. LS-SVM classifier has been widely applied in the classification of agricultural productions^41^,^44^,^45^,^46^. Proper kernel function and optimum kernel parameters are important in LS-SVM model. In this research, radial basis function (RBF) kernel function was adopted to establish the LS-SVM model. In addition, the grid-search and leave one out validation (LOOCV) methods were used to find out the optimal parameter values of regularization parameter γ and the RBF kernel function parameter σ^2^. The details of LS-SVM could be found in the literature^47^.

The shape of a receiver operating characteristics (ROC) curve and area under the curve (AUC) were often used to estimate the performance of the discrimination models. In ROC curve, parameter “Sensitivity” denotes sensitivity for each threshold value; factor “1-Specificity” stands for 1-specificity of each threshold value; parameter “area” means area under the ROC curve (AUC); and ingredient “std” represents the standard deviation of the residuals. The closer the curve is located at upper-left hand corner and the large the AUC, the better the test is at discriminating between healthy and infected petals^41^,^48^. Generally, the relation between AUC and diagnostic accuracy applies as described in Table 3^37^.

## Additional Information

**How to cite this article**: Zhao, Y.-R. *et al*. Detection of Fungus Infection on Petals of Rapeseed (*Brassica napus* L.) Using NIR Hyperspectral Imaging. *Sci. Rep.* **6**, 38878; doi: 10.1038/srep38878 (2016).

**Publisher's note:** Springer Nature remains neutral with regard to jurisdictional claims in published maps and institutional affiliations.

## Figures and Tables

**Table 1:** The one-way analysis of variance (ANOVA) statistic results of 1446 and 1656 nm.

Samples	Numbers	Mean ± S.D.^[1]^ (1446 nm)	Mean ± S.D. (1656 nm)
Healthy samples	142	0.2356 ± 0.05875^a[2]^	0.3717 ± 0.0587^a^
Infected samples	105	0.2053 ± 0.0814^b^	0.3566 ± 0.1272^a^
P value	—	<0.001	0.272

Note: [1] S. D. means Standard deviation of the group;

[2] a, b letter in the same column indicate statistical significance at the 5% level.

**Table 2:** Recognition rate (RR) of each classification model.

	Full-LS-SVM^[3]^	RF-LS-SVM^[4]^	XL-LS-SVM^[5]^	RX-LS-SVM^[6]^	1446-LS-SVM^[7]^
Variables Ratio^[9]^	238/238	4/238	2/238	6/238	1/238
Recognition rate	100.00%	83.13%	83.13%	92.68%	69.51%

Note: [3] LS-SVM established by the full wavebands (238) and categorical value; [4] LS-SVM built using the optimal wavebands (1190, 1460, 1463, and 1524 nm) selected by random frog algorithm and categorical value; [5] LS-SVM established by the optimal wavebands identified by X-loadings (1446 and 1656 nm) and categorical value; [6] LS-SVM developed by the combination of all optimal wavebands (1190, 1460, 1463, 1524, 1446, and 1656 nm) and categorical value; [7] LS-SVM established 1446 nm and categorical value, because there was significant difference at 1446 nm between healthy and infected rapeseed petals; [8] the ratio of optimal wavebands/full wavebands.

**Table 3:** Relationship between AUC and diagnostic accuracy.

Items	Evaluation Criteria					
AUC^[9]^	0.9–1.0	0.8–0.9	0.7–0.8	0.6–0.7	0.5–0.6	<0.5
Diagnostic accuracy	Excellent	Very good	Good	Sufficient	Bad	Test not useful

Note: [9] means area under the ROC curve, the closer the curve is located at upper-left hand corner and the large the AUC, the better the model is.
